# Genetically Dependent ERBB3 Expression Modulates Antigen Presenting Cell Function and Type 1 Diabetes Risk

**DOI:** 10.1371/journal.pone.0011789

**Published:** 2010-07-26

**Authors:** Hongjie Wang, Yulan Jin, M. V. Prasad Linga Reddy, Robert Podolsky, Siyang Liu, Ping Yang, Bruce Bode, John Chip Reed, R. Dennis Steed, Stephen W. Anderson, Leigh Steed, Diane Hopkins, Yihua Huang, Jin-Xiong She

**Affiliations:** 1 Center for Biotechnology and Genomic Medicine, Medical College of Georgia, Augusta, Georgia, United States of America; 2 Department of Medicine, Medical College of Georgia, Augusta, Georgia, United States of America; 3 Atlanta Diabetes Associates, Atlanta, Georgia, United States of America; 4 Southeastern Endocrine and Diabetes, Atlanta, Georgia, United States of America; 5 Pediatric Endocrine Associates, Atlanta, Georgia, United States of America; 6 Department of Pathology, Medical College of Georgia, Augusta, Georgia, United States of America; New York University, United States of America

## Abstract

Type 1 diabetes (T1D) is an autoimmune disease resulting from the complex interaction between multiple susceptibility genes, environmental factors and the immune system. Over 40 T1D susceptibility regions have been suggested by recent genome-wide association studies; however, the specific genes and their role in the disease remain elusive. The objective of this study is to identify the susceptibility gene(s) in the 12q13 region and investigate the functional link to the disease pathogenesis. A total of 19 SNPs in the 12q13 region were analyzed by the TaqMan assay for 1,434 T1D patients and 1,865 controls. Thirteen of the SNPs are associated with T1D (best p = 4×10^−11^), thus providing confirmatory evidence for at least one susceptibility gene in this region. To identify candidate genes, expression of six genes in the region was analyzed by real-time RT-PCR for PBMCs from 192 T1D patients and 192 controls. SNP genotypes in the 12q13 region are the main factors that determine *ERBB3* mRNA levels in PBMCs. The protective genotypes for T1D are associated with higher *ERBB3* mRNA level (p<10^−10^). Furthermore, ERBB3 protein is expressed on the surface of CD11c^+^ cells (dendritic cells and monocytes) in peripheral blood after stimulation with LPS, polyI:C or CpG. Subjects with protective genotypes have significantly higher percentages of ERBB3^+^ monocytes and dendritic cells (p = 1.1×10^−9^); and the percentages of ERBB3^+^ cells positively correlate with the ability of APC to stimulate T cell proliferation (R^2^ = 0.90, p<0.0001). Our results indicate that ERBB3 plays a critical role in determining APC function and potentially T1D pathogenesis.

## Introduction

With the advances in genome wide association studies (GWAS), mapping susceptibility genes involved in complex diseases has become a routine and powerful approach in elucidating the mechanisms underlying human diseases. The GWAS approach has been applied to an ever increasing number of diseases including type 1 diabetes (T1D) and type 2 diabetes among others [1]–[9]. However, an intrinsic problem with GWAS is the expected high false positive rate due to the large number of markers analyzed [10]. Therefore, putative associations identified by GWAS must be confirmed in multiple large cohorts. Further, identification of specific genes involved in the diseases remains a formidable challenge even after the genomic regions have been identified by GWAS. The challenges in the next phase of the genetic studies of complex diseases will most likely be overcome by combinatorial approaches based on genetic association and functional characterization of candidate genes located within the intervals identified by GWAS.

Several studies have reported significant associations with several SNPs in the 12q13 region [3], [7], [9], [11], [12]. These results suggested that at least one T1D susceptibility gene is located in the vicinity of these SNPs; however, the specific disease gene(s) have yet to be identified. In an effort to elucidate the specific disease genes in this region, we analyzed a large set of cases and controls from the Georgia population and defined the association interval to a genomic region of approximately 150kb. By analyzing the expression of six candidate genes in peripheral blood mononuclear cells (PBMCs) from a large batch of T1D patients and controls, the v-erb-b2 erythroblastic leukemia viral oncogene homolog 3 (avian) (*ERBB3*) gene was identified as a top candidate gene. The expression-genotype association was further confirmed by analyzing surface protein expression. Functional studies further demonstrate that ERBB3 on CD11c^+^ cells is critical to the function of antigen presenting cells (APC).

## Results

### Multiple SNPs in the 12q13 region are significantly associated with T1D

**Figure 1:**
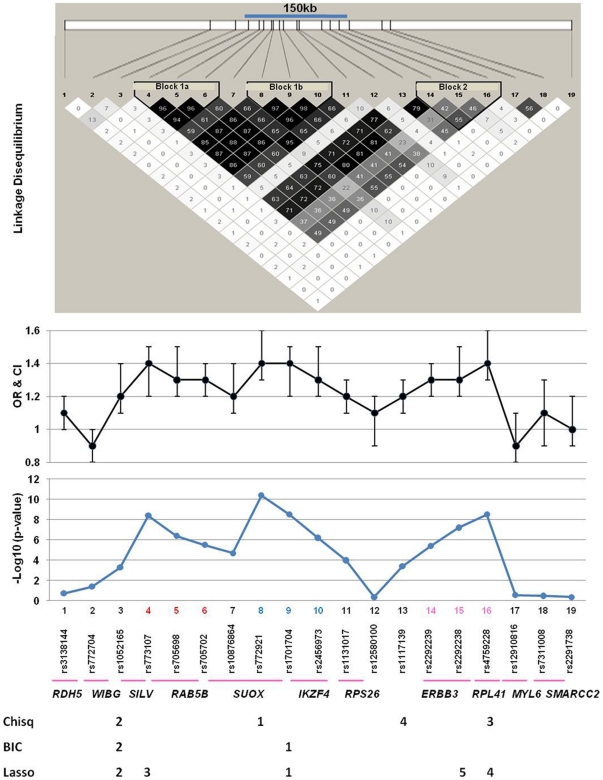
T1D is significantly associated with SNPs in the 12q13 region. Shown are LD map (top), odds ratios and 95% confidence interval (OR and CI), and −Log 10 p-values. The 150-kb region defined by SNP #4 and #16 showed some LD as indicated by the R^2^ values between pairs of SNPs. Three LD sub-blocks were identified by the HaploView 4.1 program, although there is strong LD throughout the region. The numbers below the SNPs indicate the relative importance of SNPs based on analyses aimed at determining independent effects. For the chi-square and BIC based analyses, the numbers indicate the order in which the SNP was entered in stepwise analyses. For the Lasso analysis, the number indicates the relative size of the estimated coefficient in the final model.

We genotyped 19 SNPs in the 12q13 region in 1,434 Caucasian cases and 1,865 matched controls collected in Georgia. Association between the individual SNPs and T1D was assessed using different methods as described in the Method section and presented in Table 1. Thirteen SNPs showed highly significant evidence for association. Five of the six SNPs without evidence for an association are located at the two flanking sides of the association interval and mark the approximate boundaries of the association interval (Figure 1). The SNP with the best evidence for an association (rs772921, #8) had a p-value of 4×10^−11^ and OR = 1.43 as estimated using an additive logistic regression model. Nine SNPs had p-values of <10^−5^ (Table 1). These results provide strong evidence for association between the 12q13 region and T1D in the Caucasian population in Georgia. For all SNPs, the minor allele is associated with increased risk for T1D, while the major allele is associated with lower risk. All associated SNPs are contained in a genomic region of approximately 150kb marked by SNPs rs773107 (#4) and rs4759228 (#16) (Figure 1).

**Table 1:** Association between T1D and individual SNP genotypes in the 12q13 region.

							Logistic regression additive model			
SNP **#**	SNP Name	Gene	Celera Position	Minor allele	Case freq	Cont freq	p-value	OR	LCL	UCL
1	rs3138144	RDH5	55767239	G	0.47	0.45	0.18	1.1	1.0	1.2
2	rs772704	WIBG	55968423	C	0.17	0.19	0.04	0.9	0.8	1.0
3	rs1052165	SILV	56004000	T	0.28	0.23	5×10^−4^	1.2	1.1	1.4
4	rs773107	RAB5B	56022190	G	0.38	0.31	4×10^−9^	1.4	1.2	1.5
5	rs705698	RAB5B	56037371	C	0.38	0.32	4×10^−7^	1.3	1.2	1.5
6	rs705702	SUOX	56043320	C	0.37	0.31	3×10^−6^	1.3	1.2	1.4
7	rs10876864	3′SUOX	56053768	G	0.47	0.42	2×10^−5^	1.2	1.1	1.4
8	rs772921	5′IKZF4	56056261	T	0.38	0.32	4×10^−11^	1.4	1.3	1.6
9	rs1701704	5′IKZF4	56065172	C	0.39	0.32	3×10^−9^	1.4	1.2	1.5
10	rs2456973	IKZF4	56069619	G	0.38	0.32	6×10^−7^	1.3	1.2	1.5
11	rs1131017	RPS26	56088616	C	0.46	0.41	1×10^−4^	1.2	1.1	1.3
12	rs12580100	5′ERBB3	56091897	G	0.13	0.12	0.43	1.1	0.9	1.2
13	rs11171739	5′ERBB3	56122295	C	0.42	0.38	4×10^−4^	1.2	1.1	1.3
14	rs2292239	ERBB3	56133849	A	0.38	0.32	4×10^−6^	1.3	1.2	1.4
15	rs2292238	ERBB3	56146009	G	0.46	0.39	6×10^−8^	1.3	1.2	1.5
16	rs4759228	5′RPL41	56160558	G	0.34	0.27	3×10^−9^	1.4	1.3	1.6
17	rs12810816	MYL6	56205104	G	0.10	0.11	0.27	0.9	0.8	1.1
18	rs7311008	SMARCC2	56217536	T	0.09	0.10	0.31	1.1	0.9	1.3
19	rs2291738	TIMELESS	56468655	G	0.49	0.47	0.43	1.0	0.9	1.2

OR: odds ratio, LCL: lower confidence limit, UCL: upper confidence limit.

We next tested whether the associations with the 12q13 SNPs were dependent on other covariates such as gender of the subjects, age of onset of diabetes or HLA-DQB1 genotypes using logistic regression. The 12q13 SNPs are significantly associated with T1D in both early and late onset T1D data sets with similar OR estimates (Table S1). No significant interaction was observed between SNPs and HLA genotypes (Table S2) or sex (Table S3).

### Suggestive evidence for multiple disease genes

Linkage disequilibrium (LD) estimated using the entire data set suggested LD between all SNP pairs from SNP #4 to #16 (Figure 1). However, three LD sub-blocks were suggested by the HaploView 4.1 program (Figure 1). The LD sub-block 1a includes three SNPs (#4, 5 and 6) within/near the *RAB5B* gene (R^2^ = 0.94–0.96). The LD sub-block 1b contains three SNPs (#8, 9 and 10) within/near *SUOX*/*IKZF4* (R^2^ = 0.90–0.97), while LD sub-block 2 contains three SNPs within/near *ERBB3* (#14, 15 and 16) that display weak LD (R^2^ = 0.42–0.55). Interestingly, these three LD sub-blocks coincide with the three association peaks as suggested by the plots of p-values and OR (Figure 1). Based on the LD sub-blocks, we examined associations between T1D and haplotypes within each LD block. Consistent with the strong LD in sub-block 1a (21.1kb) and 1b (13.4kb), only two major haplotypes are found in both T1D and control subjects and the haplotypes confer significantly different risk for T1D (Table 2). Significant associations were also observed between T1D and the two major haplotypes in sub-block 2 (Table 2) despite the weaker LD between the SNPs.

**Table 2:** Association between T1D and haplotypes in the 12q13 region.

LD Blocks	Haplotypes	Case freq	Ctrl freq	P-value	OR	LCI	UCI
Block 1a	ATT	0.62	0.68	2×10^−7^	0.8	0.7	0.8
SNP 4-5-6	GCC	0.37	0.31	7×10^−8^	1.3	1.2	1.5
Block 1b	CAT	0.60	0.67	2×10^−9^	0.7	0.7	0.8
SNP 8-9-10	TCG	0.39	0.32	3×10^−9^	1.4	1.2	1.5
Block 1a+1b	ATTCAT	0.593	0.661	1.4×10^−8^	0.7	0.7	0.8
	GCCTCG	0.362	0.297	4.0×10^−8^	1.3	1.2	1.5
	ATTTCG	0.018	0.015	0.2862	1.2	0.8	1.8
Block 2	AGG	0.31	0.24	1×10^−9^	1.4	1.3	1.6
SNP 14-15-16	CGG	0.01	0.01	0.4302	1.2	0.8	1.8
	CTC	0.49	0.53	2×10^−4^	0.8	0.8	0.9
	ATC	0.04	0.06	0.0190	0.8	0.6	1.0
	CTG	0.02	0.02	0.2246	0.8	0.6	1.2
	AGC	0.03	0.04	0.8087	1.0	0.7	1.3
	CGC	0.10	0.11	0.4843	0.9	0.8	1.1

Maximum-likelihood haplotype frequencies were estimated between cases and controls using Haploview version 4.1. P-values below 0.05 were considered significant and corrections were made by multiple testing (1000 permutations). LD block 2 contains the *ERBB3* gene and the strongest association is observed with a haplotype in the block “AGG”. The first 2 SNPs in this block are located at the 3′ end of ERBB3 and SNP #15 is 11kb downstream of the gene and 5′ of the RPL41 gene.

Given the LD pattern, the association observed throughout the region could be due to multiple genes. We used logistic regression analyses that included multiple SNPs to determine whether multiple SNPs have independent associations within the region. A stepwise logistic regression analysis based on the chi-squared statistic selected four SNPs (Figure 1 & Table S4): one near *SUOX* (rs772921), one within *SILV* (rs1052165), one within *RPL41* (rs4759228), and one 5′ to *ERBB3* (rs1117139). A stepwise SNP selection approach based on the Bayesian Information Criterion (BIC) selected only two SNPs: one in between *SUOX* and *IKZF4* (rs1701704) and rs1052165. Using lasso regression selected five SNPs: rs1701704, rs1052165, a SNP within RAB5B (rs773107), rs4759228, and a SNP within *ERBB3* (rs2292238). The OR estimates from the final models for the stepwise approaches were at least 1.5. The OR estimates from the lasso analysis were smaller due to the shrinkage used. Only two of the SNPs had an OR of at least 1.2 based on this analysis (rs1701704 and rs1052165). These results suggest that the association observed within this region may be due to multiple genes. However, the precise number, location and identity of the disease genes could not be determined using the genetic association data.

### SNP association with *ERBB3* mRNA levels in PBMCs

**Figure 2:**
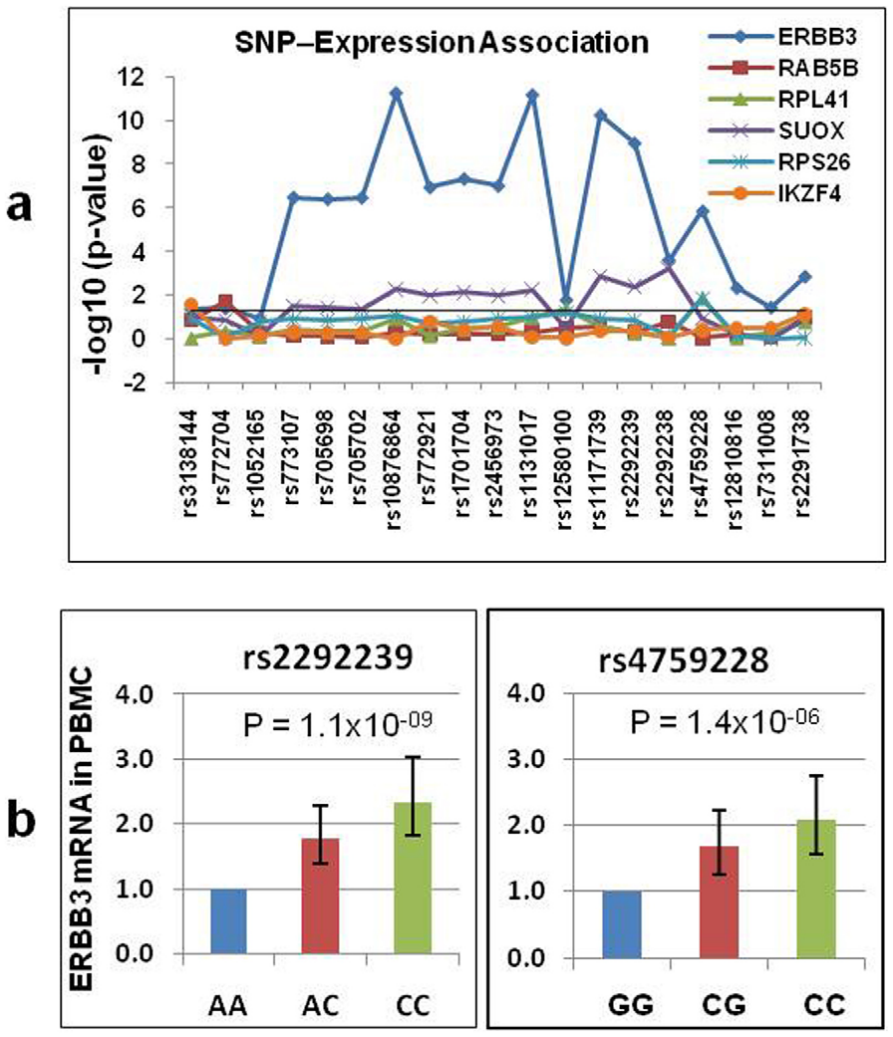
Evidence for association between SNPs and *ERBB3* gene expression in PBMCs. (**a**) Associations between each of the 19 SNPs and the expression of each of the 12q13 genes were assessed by regression that included the additive effect of each SNP and T1D status. The −Log10 p-value for each comparison was plotted against the genomic order of the 19 SNPs. *ERBB3* expression showed strong association with multiple SNPs including one SNP in the middle (rs2292239) and one SNP at the 3′ end (rs4759228) of the *ERBB3* gene. (**b**) The mean expression of *ERBB3* was presented for the three genotypes at rs2292239 and rs4759228, respectively. The mean expression level was arbitrarily set to 1 for the minor allele homozygous genotype at each SNP. P-values are for the additive effect of the SNP in a regression model.

Since the association data did not allow us to identify the most likely candidate SNPs or genes, we searched for potential association between genotype and gene expression to identify candidate genes. The expression of six genes (*RAB5B*, *SUOX*, *IKZF4*, *RPS26*, *RPL41*, and *ERBB3*) located in the 12q13 association interval were analyzed in 384 subjects (192 T1D patients and 192 controls with similar age and sex distribution). The HLA genotypes were not matched between cases and controls. All genes were detectable in PBMCs using real-time RT-PCR. Regression modeling was used to examine the relationship between SNP genotype and gene expression using T1D status as a covariate. These analyses indicated that the expression levels for four genes (*RAB5B*, *IKZF4*, *RPS26* and *RPL41*) did not depend on the genotypes in the 12q13 region (Figure 2a). IKZF4 is an excellent candidate for T1D susceptibility gene in the region because it is important for the function of regulatory T cells [13]. While our data cannot exclude *IKZF4* as a candidate gene, the lack of expression-genotype correlation decreased the priority for this gene in our subsequent studies. Previous reports based on genome-wide expression and SNP data [14]–[17] suggested that *RPS26* may be a good candidate gene. Our inability to confirm this finding decreased the priority for subsequent studies. *SUOX* mRNA levels showed marginally significant association with several SNPs and should be further investigated in future studies. However, the most interesting finding from the gene expression studies was on *ERBB3* which showed a highly significant (p<10^−10^) association between expression levels and genotypes at multiple SNPs (Figure 2a).

A Student t-test also suggests that the mRNA levels of *ERBB3* differ by more than two-fold between the homozygous genotypes at rs2292239 (SNP #14) and rs4759228 (SNP #16) (Figure 2b). These analyses indicate that *ERBB3* mRNA levels are significantly lower (p = 1.1×10^−9^ and 1.4×10^−6^, respectively) for the minor allele homozygotes (susceptible genotype) at rs2292239 and rs4759228 (Figure 2b), suggesting that reduced *ERBB3* expression is associated with genotypes that confer increased risk for T1D.

### Up-regulation of surface ERBB3 in CD11c^+^ cells by infection mimics

**Figure 3:**
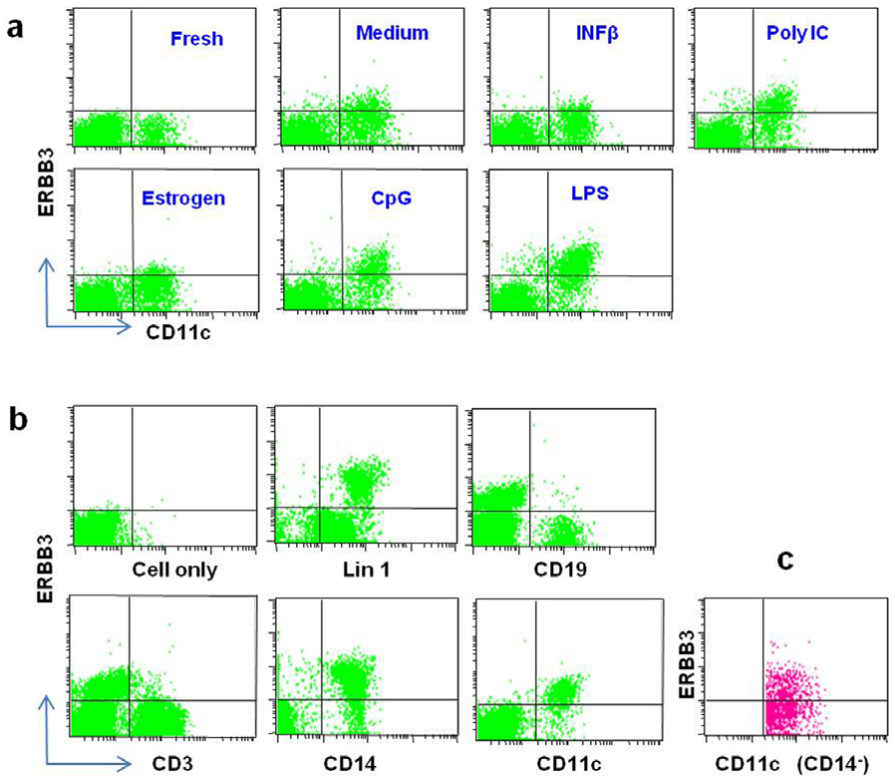
ERBB3 protein expression on the surface of PBMCs. (**a**) Representative FACS profiles for surface ERBB3. All panels are dual staining for ERBB3 and CD11c. The left top panel (Fresh) is an example for staining of freshly isolated PBMCs. All other panels are staining for PBMCs that were cultured for 24 hours with one of the five stimuli or medium alone. (**b**) Staining for ERBB3 in different lymphocyte subsets in PBMCs. With the exception of “cell only”, all panels were stained for ERBB3 and another antibody labeled underneath the panel. (**c**) Three-color staining of PBMCs for ERBB3, CD11c and CD14. Shown is ERBB3 expression on dendritic cells (CD11c^+^CD14^−^).

This study is the first to detect expression of *ERBB3* in the PMBC. However, the immune cell type(s) that express *ERBB3* were not identified by the gene expression studies. To identify the specific cell types of PBMC that express ERBB3, we evaluated several monoclonal antibodies and identified one clone that can be used for FACS analysis. Surface ERBB3 was almost undetectable in fresh PBMCs (Figure 3a); however, surface ERBB3 protein was dramatically increased after cultures with three different infection mimics (LPS, polyI:C and CpG) (Figure 3a). Co-staining of PBMC with the anti-ERBB3 antibody and cell-type-specific antibodies indicated that almost all ERBB3^+^ PBMCs express CD11c, a marker for monocytes and dendritic cells (DC), and CD14, a monocyte marker; while few T cells (CD3^+^) and B cells (CD19^+^) are positive for ERBB3 on the cell surface (Figure 3b). These results suggest that monocytes express surface ERBB3. To determine whether DC also expresses ERBB3, we co-stained PBMCs with antibodies against ERBB3, CD11c and CD14. As shown in Figure 3c, a subset of DC (CD11c^+^CD14^−^ cells) also expresses ERBB3 after treatment with the three infection mimics.

### Genotype-dependent ERBB3 surface expression

**Figure 4:**
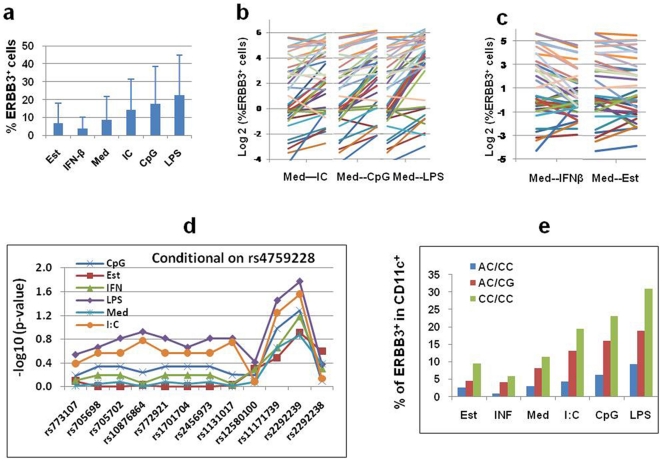
Surface ERBB3 expression depends on the types of stimulation and SNP genotypes. (**a**) Mean percentage of ERBB3^+^ cells among CD11c^+^ PBMCs after culture with medium alone (Med) or different stimulation: Est (estrogen), IFN-β, IC (polyI:C), CpG and LPS. (**b**) Inter-individual variation of percentage of ERBB3^+^ cells among CD11c^+^ PBMCs after stimulation. Data were Log 2 transformed. Data points for cultures with medium alone (Med) and one stimulation (IC, CpG or LPS) in each individual were linked to illustrate the changes in ERBB3 under different culture conditions. (**c**) The impact of IFN-β and estrogen on ERBB3 expression. The data are presented in similar ways as in (**b**). (**d**) The association between percent of ERBB3^+^ cells in CD11c^+^ PBMCs and SNP pairs. Regression was used to examine whether two SNPs could improve the association between % of ERBB3^+^ cells and genotype. The −Log10 p-value for each SNP added to a model that already included SNP rs4759228 was plotted against the genomic order of the SNPs. These results indicate that the percentages of ERBB3^+^ cells are partially determined by genotypes within/near the *ERBB3* gene. (**e**) Mean percentages of ERBB3^+^ cells were presented according to culture conditions and two-locus genotypes at rs2292239 and rs4759228.

During our studies of ERBB3 surface expression, we observed individual variation in surface ERBB3 after *in vitro* treatment with infection mimics. Therefore, we expanded our studies to 39 randomly selected control subjects. After culture of the PBMCs with various conditions, the cells were stained with anti-ERBB3 and anti-CD11c antibodies. The results revealed high inter-individual variability (Figure 4a, 4b & 4c). For example, 46%, 23% and 31% of the subjects have high (18–79%, mean = 42%), medium (8–16%, mean = 12%) and low (<1.6%, mean = 0.7%) percentages of ERBB3^+^ cells after 24 hour stimulation with LPS. Furthermore, after LPS, CpG or polyI:C treatment, there were high percentages of ERBB3^+^ cells (Figure 4b). In contrast, treatment with IFN-β or estrogen do not have a significant role in inducing ERBB3 compared to medium (Figure 4c).

To further elucidate the mechanism underlying the individual variation in surface ERBB3 expression, we examined the association between the percentages of ERBB3^+^ cells and a number of variables including, sex, age and *ERBB3* genotypes. Consistent with the association between *ERBB3* mRNA levels and SNP genotypes discussed earlier (Figure 2), the percentages of ERBB3^+^ cells after stimulation differed between *ERBB3* genotypes at multiple SNPs including rs2292239 and rs4759228 (data not shown). We then used regression analysis to examine whether two SNPs could improve the association between genotypes and the percentages of ERBB3^+^ cells. For these analyses, we included a single SNP in LD sub-block 2, one at a time, and then added the remaining SNPs, one at a time. The results indicate that the additive effects of both rs2292239 and rs4759228 contribute to differences in ERBB3 expression (Figure 4d). Therefore, we calculated the mean percentages of ERBB3^+^ cells for three two-locus genotypes (AC/CC, AC/CG and CC/CC). The CC/CC genotype, composed of the protective CC homozygote at rs2292239 and the protective CC homozygote at rs4759228, had the highest mean percentages of ERBB3^+^ cells. The differences between these two-locus genotypes were quite large and consistent across all culture conditions (Figure 4e). These results for the ERBB3 surface protein, together with the data on *ERBB3* mRNA, provide strong evidence that *ERBB3* gene and surface protein expression is genotype-dependent.

### The percentages of ERBB3^+^ cells correlate with APC function

**Figure 5:**
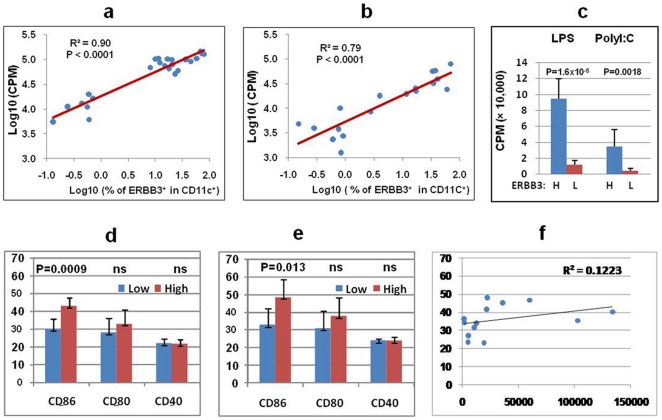
Impact of ERBB3 on APC function. (**a**) Correlation between APC function (ability to stimulate T cell proliferation) and percentages of ERBB3^+^ cells of the APC source. Each dot represents one individual plotted based on their percentage of ERBB3^+^ cells after LPS stimulation and the associated proliferation. (b) Correlation between APC function and ERBB3 after polyI:C stimulation. (**c**) Association between T cell proliferation and percentage of ERBB3^+^ cells of the APC source. PBMCs were cultured for 24 hours with LPS or polyI:C and then irradiated and used as APC to stimulate the proliferation of responder T cells purified from one healthy donor. The mean proliferation (CPM) was calculated for subjects with high ERBB3 (H) defined as having >10% ERBB3^+^ cells among CD11c^+^ PBMCs and low ERBB3 (L) defined as having <1% ERBB3^+^ cells. Student t-test was used to assess statistical significance. (**d**) FACS analysis of CD86, CD80 and CD40 on PBMCs after *in vitro* culture. The means and standard deviations (mean fluorescent intensity = MFI) for subjects with low or high proliferation were presented for CD86, CD80 and CD40. Cells were stimulated with LPS. (**e**) Expression levels of CD86, CD80 and CD40 after polyI:C stimulation. Results were presented as described in (d). (**f**) Correlation between CD86 expression (MFI on the Y axis) and responder T cell proliferation (CPM on the X axis).

Since ERBB3 is mainly expressed in CD11c^+^ antigen presenting cells, we hypothesized that ERBB3 may play an important role in APC function. This hypothesis was first tested by evaluating the ability of APC with different percentages of ERBB3^+^ cells to stimulate T cell proliferation. To avoid variation from responder T cells, we purified responder CD4^+^ T cells from one single healthy donor. The responder CD4^+^ T cells were stimulated by irradiated PBMCs from different subjects who have different percentages of ERBB3^+^ cells after stimulation with LPS or polyI:C. The proliferation of the responder T cells was highly variable and ranged from 1,000 CPM to 100,000 CPM among the tested PBMCs. Consistent with our hypothesis, regression analyses suggested that the proliferation of responder T cells was significantly associated with the percentages of ERBB3^+^ cells of the APC source (p<0.0001, Figure 5a & 5b). APC from subjects with high percentages (>10%) of ERBB3^+^ cells had an 8-fold higher stimulation capacity than APC from subjects with low percentages (<1%) of ERBB3^+^ cells (p = 1.6×10^−8^, Figure 5c). The stimulation capacity of APC is potentially influenced by many factors including HLA and costimulatory molecules. The HLA genotypes were not matched between subjects with low and high percentages of ERBB3^+^ cells. However, there was no significant association between HLA and the stimulation capacity of the APC (data not shown). This result is not surprising because our stimulation condition contains anti-CD3 and anti-CD28 antibodies which can minimize differences due to HLA. We also measured the expression levels of three costimulatory molecules (CD86, CD80 or CD40). There was no difference in CD40 expression between APC with high and low stimulation activity (Figure 5d & 5e). CD80 expression was slightly but not significantly higher in APC with high stimulation activity than APC with low stimulation activity (Figure 5d & 5e). Interestingly, the mean CD86 level is significantly higher in APC with high stimulation activity than APC with low stimulation activity in both LPS stimulated cells (Figure 5d, p = 0.0009) and polyI:C stimulated cells (Figure 5e, p = 0.01). However, the correlation between CD86 levels and the stimulation function of APC is very poor (R^2^<0.2, Figure 5f). Therefore, CD86 cannot explain the correlation between ERBB3 and APC's stimulation function.

## Discussion

This study provided strong evidence for association between T1D and multiple SNPs and haplotypes in the 12q13 region in the Georgia Caucasian population. Together with the results from the published reports, there is now convincing evidence for this association interval. Based on our genetic association data, thirteen SNPs located within an expanded 150kb genomic region showed strong evidence for association. Although associations across large genomic regions could be explained by LD between SNPs, our results suggested the possibility of multiple T1D genes in this 12q13 region. However, the genetic mapping data in this study or in previous studies did not provide conclusive evidence that allows the identification of specific SNPs or genes responsible for the observed association. Several previous genome-wide studies on SNP-gene expression association identified a significant association between the *RPS26* gene in the 12q13 region and SNPs within *ERBB3* and near *RPS26* in B cell lines [16], [17], liver [14], brain and PBMC [14], [15]. It has been suggested that *RPS26* is a strong candidate gene for the observed genetic association with T1D. This study, with a large sample size, did not find any evidence for association between *RPS26* expression and SNP genotypes. Furthermore, a previous report suggested that *RPS26* expression is unlikely to be the molecular phenotype responsible for T1D susceptibility in this region [18]. Given the controversial conclusions in different studies and the lack of functional data, we believe that additional studies are required to determine whether *RPS26* is a T1D susceptibility gene.

In this study, our real-time RT-PCR analysis of gene expression in PBMC identified two possible genes with significant genotype-expression association. While *SUOX* showed a genotype-expression association and should be further studied for its potential role in T1D, *ERBB3* stood out as the most promising candidate gene for T1D due to the observed highly significant genotype-expression association. Therefore, our subsequent efforts focused on *ERBB3*. Although genotype-expression association and genotype-disease association together are important indications for disease genes, such evidence alone is not definitive proof for causality. In addition to the genetic and functional associations, the candidate gene must be functionally relevant to the disease pathogenesis. *ERBB3* encodes a member of the epidermal growth factor receptor (EGFR) family of receptor tyrosine kinases [19]. It is a membrane-bound protein with a neuregulin binding domain but without an active kinase domain [20]. It can bind its ligand without conveying the signal into the cell through protein phosphorylation. However, the heterodimers formed with other EGF receptor family members that do have kinase activity lead to the activation of pathways involved in cell proliferation or differentiation. *ERBB3* is widely known for its role in cancer as amplification of this gene and/or over-expression of its protein have been shown in numerous cancers, including prostate, bladder, gastric cancer, and breast tumors [21]–[25]. *ERBB3* is suspected to be a good candidate gene for T1D because it is known to be expressed in pancreatic beta cells and ductal cells [26], [27].

The current study suggests for the first time that *ERBB3* plays a critical role in immune regulation. We have demonstrated that ERBB3 is expressed on the surface of CD11c^+^ cells (monocytes and DC) upon *in vitro* stimulation by infection mimics, suggesting that ERBB3 may play an important role in innate immune response. Consistent with this view, we have shown that the ability of APC to stimulate T cell proliferation is highly significantly correlated with the percentage of ERBB3^+^ cells. However, how ERBB3 modulates APC function requires further investigation. As an EGF family receptor, ERBB3 and its heterodimeric partners are known to influence the expression of a large number of genes including growth factors, cytokines and transcriptional control [28], which play critical roles in immune response and regulation. ERBB3 is critical to the activation of the ERBB3-PI3K-Akt cascade induced by EGFR/ERBB3 heterodimers [29]. Deficiency or inhibition of the PI3K pathway can lead to reduced numbers of regulatory T cells and autoimmunity [30].

Our data suggest that the percentage of ERBB3^+^ cells is correlated with the ability of APC to stimulate T cell proliferation. In our study, we assessed the proliferation of the same CD4^+^ T cells responding to APC from individuals with different genetic backgrounds and levels of ERBB3 expression. It is possible that different HLA genes in those individuals may also contribute to the varying degree of proliferation of responder T cells. However, the influence of HLA heterogeneity is minimized by adding anti-CD3 and anti-CD28 antibodies, and HLA could not explain the observed differences in APC function. LPS, CpG and others stimuli are known to change the expression profiles of many other genes in addition to ERBB3, which may affect the antigen presenting function of APCs. Indeed, our data suggest that CD86 expression levels may be partly responsible for the differences in APC function. However, CD86 cannot explain the correlation observed between ERBB3 and APC function. It remains unknown whether ERBB3 is directly implicated in the stimulation of T cell proliferation. Based on the known function of ERBB3, it is more likely that the expression of ERBB3 may influence the gene expression profiles in APC and therefore indirectly modulate APC function. APC can influence susceptibility to autoimmunity through different mechanisms including deletion of autoreactive T cells, activation of regulatory T cells and immune deviation. Irrespective of the precise immunological mechanisms, our study suggests that ERBB3 may be an important molecular link between genetic susceptibility, infection and the adaptive immune response, which are all believed to contribute to T1D pathogenesis.

## Methods

### Ethics statement

This study was approved by the Medical College of Georgia Institutional review Board. All patients provided written informed consent for participation in the study and donation of samples.

### Patient population

The study population consists of Caucasian T1D patients and healthy controls from Georgia and has been used in previous studies [31]. The data set includes 1,434 patients and 1,865 controls. All diabetic patients were diagnosed using the criteria of the American Diabetes Association. Most of the cases (893) developed T1D at or before the age of 17 years (referred to as early onset), while 541patients developed diabetes after 17 years of age (referred to as late onset). The normal controls do not have a family history for T1D and were ascertained from the same geographic area as the patients.

### Genotyping

The SNPs were genotyped using TaqMan-assays designed and validated by Applied Biosystems [32]. Amplification reactions were performed in a 5 µl final volume in optical 384-well plates. PCR was carried out with 2 min at 50°C, 10 min at 95°C followed by 40 cycles of 15 sec at 95°C and 1 min at 60°C using an ABI 7900HT Fast Real-Time PCR system (Applied Biosystems).

### Gene expression assays by real-time RT-PCR

Peripheral blood was immediately preserved in the PaxGene RNA tubes. After two hours at room temperature, the tubes were frozen at −80°C freezers and total RNA was extracted within a few weeks using a Qiagen kit specially developed for the PaxGene RNA tubes. Extracted RNA was stored at −80°C freezers till use. An aliquot of 2ug RNA were arrayed in 96-well plates and then converted to cDNA using a High Capacity cDNA Reverse Transcription Kit (Applied Biosystems). The cDNA products are diluted and an aliquot of cDNA equivalent of 10ng total RNA was used for quantitative real-time PCR performed using TaqMan assays from the Applied Biosystems. Assays include *ERBB3* (Hs00176538_m1), *RAB5B* (Hs00161184_m1), *SUOX* (Hs00166578_m1), *RPL41* (Hs00606029_g1), *RPS26* (Hs00762561_s1) and *IKZF4* (Hs00223842_m1). The *18s rRNA* (Hs 99999901_s1) and *GAPDH* (Hs99999905_m1) were used as endogenous control for normalizing RNA concentration. Real-time PCR was performed in 384-well plates with the ABI 7900HT Fast Real-Time PCR System. Standard thermal cycling condition (10 min at 95°C, 40 cycles for 15 sec at 95°C, 1 min at 60°C) was used for all genes. A total of 384 samples (192 T1D and 192 controls) were analyzed. Cycle threshold (C_T_) values for each gene were obtained for each sample using the SDS2.3 and analyzed with RQ Manager 1.2 software. Differences in C_T_ values between a test gene and endogenous controls (18s rRNA and GAPDH) (ΔC_T_) were calculated and used for statistical analyses.

### Cell culture

PBMCs were isolated by Ficoll-paque density gradient centrifugation. Cells were cultured in RPMI 1640 medium (Cellgro, VA) with 10% FBS (Sigma, MO), 5 mM HEPES (Fisher Scientific, NJ), penicillin (50 µg/ml), streptomycin (50 µg/ml), 1mM sodium pyruvate (Cellgro, VA)) and 50 µM 2-mercaptoethanol (Invitrogen, NY) in 24 and 96-well plates (Costa, Cambridge, MA). For stimulation, 1×10^6^ cells were cultured in 24-well plate and then treated with 1 µg/ml LPS (Sigma, MO), 0.5 µM CpG (ODN2006, AXXORA, CA), 1 µg/ml estrogen (Sigma, MO), 300IU/ml IFN-β (Sigma, MO) and 12.5 µg/ml poly(I:C) (Sigma, MO) for 24h.

### FACS analysis

For each staining, 5×10^5^ PBMCs were resuspended in 100µl PBS buffer, blocked with human FcR blocking reagent (Miltenyi) for 10 min, then stained with antibodies: APC-anti-CD11c (clone: B-ly6, BD PharMingen), PE anti-ERBB3 (clone:66223, R&D Systems), APC-anti-CD19 (BD PharMingen), FITC-anti-CD14 (BD PharMingen) and FITC-Lin 1 (CD3, CD14, CD16, CD19, CD20 and CD56) (BD Biosciences) and then incubated on ice in the dark for 40 min. 7-AAD was used for gating out dead cells. For cell subset analysis, 2.5×10^6^ cells were used. Background fluorescence was assessed using appropriate isotype antibodies. Fluorescence was quantified using a FACS Calibur flow cytometer (BD Biosciences). Cells were electronically gated on forward angle light scatter to exclude contaminating erythrocytes and small debris and on 90° light scatter to exclude granulocytes. Data were analyzed using Cell Quest.

### T cell stimulation assay

Peripheral blood (∼20 ml) was collected in sodium-heparin vacutainer tubes from one single healthy donor. CD4^+^ T cell population was isolated using a human CD4^+^ T cells enrichment cocktail (Stem Cell). CD4^+^ T cells (5×10^4^ or 2.5×10^4^ cells per well) were co-cultured with irradiated PBMC (1×10^5^ or 0.5×10^5^ cells per well) after culture with LPS or polyI:C. A stimulus of 0.5 or 0.25 µg/ml soluble anti-CD3 (clone HIT3a) and 0.25 or 0.125 µg/ml soluble anti-CD28 (clone CD28.2; BD Pharmingen) were added in the co-culture medium. After 48h, 0.5 µCi ^3^H-thymidine (Amersham Biosciences, Piscataway, NJ) was added for the final 16h of culture to assess CD4^+^ T cell proliferation.

### Statistical analysis for SNP association

Association between each SNP and T1D status was assessed using different methods. Since the results were very similar, we only presented the data from logistic regression with an additive model. We examined the potential effects of sex and HLA risk by including these variables as covariates in the additive genetic model of a logistic regression. We used the Breslow-Day test to examine heterogeneity in the ORs between low- and high-risk HLA-DQB1 subsets. The early and late onset patients were analyzed separately and jointly to examine the potential influence of age of onset on the SNP associations. Given the strong signal observed throughout the region and the strong linkage disequilibrium in this region, we used logistic regression as the basis for determining which SNPs showed independent effects. Two stepwise approaches and one model selection/shrinkage approach were used for this purpose [33]. In all models, we used additive SNP effects with no interactions. In the first approach, we sequentially added SNPs to the model based on the largest chi-squared test for the added SNP, until no additional SNP was significant. In this approach, we used all of the samples to increase power. Sample sizes decreased as SNPs were added due to subjects having incomplete genotype information for all SNPs. For the other two approaches, we only used the data for subjects having complete genotype data for all SNPs. In the second approach, we used the Bayes Information Criterion (BIC) to add SNPs. The single SNP model with the smallest BIC was chosen in the first step, with subsequent SNPs being added based on the model having the smallest BIC, provided the BIC was smaller than the accepted model from the previous step. The third approach that we used was lasso-based shrinkage.

### Statistical analysis for gene expression and FACS data

Gene expression differences between SNP genotypes were examined by student t-test in some analyses and by a regression model in which the association between gene expression and SNP genotype was additive, which is equivalent to asking whether a trend exists in the association between gene expression and SNP genotype. In such models, the estimated mean for the heterozygote will be equal to the average of the homozygotes. Analyses to examine the potential role of multiple SNPs utilized models that included two SNPs, each with an additive effect. These models also included the effect of T1D status and all interaction. All mixed-model analyses were conducted using the Proc Mixed procedure of SAS, and models without random effects were conducted using the Proc GLM procedure.

FACS data were analyzed using student t-tests and regression modeling. The differences between genotypes were examined using an additive model, similar to the analyses conducted for the expression data. We initially included the additive effects of two SNPs in one model to examine the potential role of multiple loci. Since not all two-locus genotypes were observed in our study, we also examined only those two locus genotypes having at least five subjects. For this case, we used the effect of two-locus genotype in a one-way ANOVA.

## Supporting Information

Table S1 Association in early and late onset T1D patients. *Logistic regression additive model.
(0.05 MB DOC)

Table S2 Association between T1D and 12q13 SNPs with HLA as a covariate. *Logistic regression additive model.
(0.05 MB DOC)

Table S3 Association between T1D and 12q13 SNPs with sex as a covariate. *Logistic regression additive model.
(0.05 MB DOC)

Table S4 Results of multiple-SNP logistic regression analyses aimed at determining which SNPs had independent effects.
(0.04 MB DOC)
