# Supplementary material for: Genetically Dependent ERBB3 Expression Modulates Antigen Presenting Cell Function and Type 1 Diabetes Risk
Source: PLoS One. 2010 Jul 26;5(7):e11789. doi: 10.1371/journal.pone.0011789 (PMC2909911; doi:10.1371/journal.pone.0011789)
Supplement: Table S1 — Association in early and late onset T1D patients. *Logistic regression additive model. (0.05 MB DOC) [file pone.0011789.s001.doc]

**Table S1**

| SNP | SNP Name | Early onset *Additive | | | |  | Late onset * Additive Heterogenity | | | | |
| --- | --- | --- | --- | --- | --- | --- | --- | --- | --- | --- | --- |
| Number |  | OR | LCL | UCL | p-value |  | OR | LCL | UCL | p-value | P-value |
| 1 | rs3138144 | 1.1 | 1.0 | 1.2 | 0.1023 |  | 1.0 | 0.9 | 1.2 | 0.8323 | 0.6032 |
| 2 | rs772704 | 0.9 | 0.8 | 1.0 | 0.1354 |  | 0.8 | 0.7 | 1.0 | 0.0561 | 0.9848 |
| 3 | rs1052165 | 1.3 | 1.1 | 1.5 | 0.0001 |  | 1.1 | 1.0 | 1.3 | 0.1159 | 0.2807 |
| 4 | rs773107 | 1.4 | 1.2 | 1.5 | 5x10^-7^ |  | 1.4 | 1.2 | 1.6 | 4x10^-5^ | 0.7411 |
| 5 | rs705698 | 1.3 | 1.2 | 1.5 | 2x10^-5^ |  | 1.3 | 1.1 | 1.5 | 0.0003 | 0.9656 |
| 6 | rs705702 | 1.3 | 1.1 | 1.4 | 0.0002 |  | 1.3 | 1.1 | 1.5 | 0.0003 | 0.9788 |
| 7 | rs10876864 | 1.3 | 1.1 | 1.4 | 2x10^-5^ |  | 1.2 | 1.0 | 1.3 | 0.0304 | 0.1091 |
| 8 | rs772921 | 1.4 | 1.3 | 1.6 | 5x10^-9^ |  | 1.4 | 1.2 | 1.6 | 9x10^-6^ | 0.3378 |
| 9 | rs1701704 | 1.4 | 1.2 | 1.5 | 3x10^-7^ |  | 1.4 | 1.2 | 1.6 | 2x10^-5^ | 0.6421 |
| 10 | rs2456973 | 1.3 | 1.1 | 1.5 | 4x10^-5^ |  | 1.3 | 1.1 | 1.5 | 0.0001 | 0.9525 |
| 11 | rs1131017 | 1.2 | 1.1 | 1.4 | 0.0007 |  | 1.2 | 1.0 | 1.4 | 0.018 | 0.4469 |
| 12 | rs12580100 | 1.0 | 0.9 | 1.2 | 0.9426 |  | 1.2 | 1.0 | 1.4 | 0.1442 | 0.5331 |
| 13 | rs11171739 | 1.2 | 1.1 | 1.3 | 0.0013 |  | 1.2 | 1.0 | 1.3 | 0.0308 | 0.4797 |
| 14 | rs2292239 | 1.3 | 1.1 | 1.4 | 0.0001 |  | 1.3 | 1.1 | 1.5 | 0.0015 | 0.6651 |
| 15 | rs2292238 | 1.3 | 1.2 | 1.5 | 2x10^-6^ |  | 1.3 | 1.1 | 1.5 | 0.0003 | 0.7911 |
| 16 | rs4759228 | 1.4 | 1.2 | 1.6 | 2x10^-7^ |  | 1.4 | 1.2 | 1.6 | 3x10^-5^ | 0.5294 |
| 17 | rs12810816 | 0.9 | 0.8 | 1.1 | 0.5097 |  | 0.9 | 0.7 | 1.1 | 0.1781 | 0.6510 |
| 18 | rs7311008 | 1.1 | 0.9 | 1.3 | 0.6317 |  | 1.2 | 0.9 | 1.5 | 0.1583 | 0.7197 |
| 19 | rs2291738 | 1.0 | 0.9 | 1.1 | 0.8474 |  | 1.1 | 1.0 | 1.3 | 0.2201 | 0.3130 |
