# Supplementary material for: Genetically Dependent ERBB3 Expression Modulates Antigen Presenting Cell Function and Type 1 Diabetes Risk
Source: PLoS One. 2010 Jul 26;5(7):e11789. doi: 10.1371/journal.pone.0011789 (PMC2909911; doi:10.1371/journal.pone.0011789)
Supplement: Table S2 — Association between T1D and 12q13 SNPs with HLA as a covariate. *Logistic regression additive model. (0.05 MB DOC) [file pone.0011789.s002.doc]

**Table S2**

| SNP | SNP Name | Low Risk *Additive | | | |  | High Risk *Additive | | | | |
| --- | --- | --- | --- | --- | --- | --- | --- | --- | --- | --- | --- |
| Number |  | OR | LCL | UCL | p-value |  | OR | LCL | UCL | p-value | Heterogenity  P-value |
| 1 | rs3138144 | 1.1 | 1.0 | 1.2 | 0.1789 |  | 1.3 | 1.0 | 1.6 | 0.0521 | 0.29 |
| 2 | rs772704 | 0.8 | 0.7 | 1.0 | 0.0356 |  | 0.9 | 0.7 | 1.2 | 0.4281 | 0.15 |
| 3 | rs1052165 | 1.2 | 1.0 | 1.4 | 0.0185 |  | 1.5 | 1.1 | 2.0 | 0.0118 | 0.84 |
| 4 | rs773107 | 1.3 | 1.1 | 1.5 | 0.0002 |  | 1.4 | 1.1 | 1.8 | 0.0058 | 0.62 |
| 5 | rs705698 | 1.3 | 1.1 | 1.4 | 0.0010 |  | 1.3 | 1.0 | 1.6 | 0.0528 | 0.94 |
| 6 | rs705702 | 1.3 | 1.1 | 1.4 | 0.0012 |  | 1.4 | 1.1 | 1.8 | 0.0086 | 0.48 |
| 7 | rs10876864 | 1.2 | 1.1 | 1.4 | 0.0027 |  | 1.3 | 1.0 | 1.6 | 0.0389 | 0.74 |
| 8 | rs772921 | 1.4 | 1.2 | 1.6 | 4x10^-6^ |  | 1.5 | 1.2 | 1.9 | 0.0014 | 0.84 |
| 9 | rs1701704 | 1.3 | 1.2 | 1.5 | 3x10^-5^ |  | 1.4 | 1.1 | 1.8 | 0.0086 | 0.98 |
| 10 | rs2456973 | 1.3 | 1.1 | 1.5 | 0.0002 |  | 1.3 | 1.0 | 1.6 | 0.0614 | 0.70 |
| 11 | rs1131017 | 1.2 | 1.1 | 1.4 | 0.0045 |  | 1.4 | 1.1 | 1.8 | 0.0066 | 0.33 |
| 12 | rs12580100 | 1.0 | 0.8 | 1.2 | 0.9488 |  | 1.2 | 0.8 | 1.7 | 0.3133 | 0.60 |
| 13 | rs11171739 | 1.2 | 1.1 | 1.4 | 0.0055 |  | 1.3 | 1.0 | 1.6 | 0.0576 | 0.83 |
| 14 | rs2292239 | 1.2 | 1.1 | 1.4 | 0.0017 |  | 1.3 | 1.1 | 1.7 | 0.0172 | 0.67 |
| 15 | rs2292238 | 1.3 | 1.1 | 1.5 | 0.0001 |  | 1.3 | 1.1 | 1.7 | 0.0172 | 0.76 |
| 16 | rs4759228 | 1.5 | 1.3 | 1.7 | 2x10^-7^ |  | 1.3 | 1.0 | 1.6 | 0.0693 | 0.42 |
| 17 | rs12810816 | 0.9 | 0.8 | 1.2 | 0.5919 |  | 0.8 | 0.5 | 1.1 | 0.1507 | 0.57 |
| 18 | rs7311008 | 1.1 | 0.9 | 1.3 | 0.5493 |  | 1.2 | 0.8 | 1.8 | 0.3364 | 0.46 |
| 19 | rs2291738 | 1.0 | 0.9 | 1.2 | 0.6455 |  | 1.1 | 0.9 | 1.4 | 0.5367 | 0.73 |
