# Supplementary material for: Genetically Dependent ERBB3 Expression Modulates Antigen Presenting Cell Function and Type 1 Diabetes Risk
Source: PLoS One. 2010 Jul 26;5(7):e11789. doi: 10.1371/journal.pone.0011789 (PMC2909911; doi:10.1371/journal.pone.0011789)
Supplement: Table S3 — Association between T1D and 12q13 SNPs with sex as a covariate. *Logistic regression additive model. (0.05 MB DOC) [file pone.0011789.s003.doc]

**Table S3**

| SNP | SNP Name | Males *Additive | | | |  | Females *Additive | | | | |
| --- | --- | --- | --- | --- | --- | --- | --- | --- | --- | --- | --- |
| Number |  | OR | LCL | UCL | p-value |  | OR | LCL | UCL | p-value | Heterogenity  P-value |
| 1 | rs3138144 | 1.0 | 0.8 | 1.1 | 0.5706 |  | 1.1 | 1.0 | 1.3 | 0.1717 | 0.6000 |
| 2 | rs772704 | 0.9 | 0.7 | 1.1 | 0.2082 |  | 0.9 | 0.7 | 1.0 | 0.1015 | 0.9705 |
| 3 | rs1052165 | 1.3 | 1.1 | 1.5 | 0.0036 |  | 1.2 | 1.0 | 1.4 | 0.0281 | 0.6134 |
| 4 | rs773107 | 1.3 | 1.2 | 1.5 | 0.0001 |  | 1.4 | 1.2 | 1.6 | 1x10^-5^ | 0.5456 |
| 5 | rs705698 | 1.3 | 1.1 | 1.5 | 0.0009 |  | 1.3 | 1.1 | 1.5 | 0.0002 | 0.6796 |
| 6 | rs705702 | 1.2 | 1.0 | 1.4 | 0.0108 |  | 1.4 | 1.2 | 1.6 | 7x10^-5^ | 0.2248 |
| 7 | rs10876864 | 1.2 | 1.1 | 1.4 | 0.0027 |  | 1.3 | 1.1 | 1.4 | 0.0015 | 0.9360 |
| 8 | rs772921 | 1.4 | 1.2 | 1.6 | 3x10^-5^ |  | 1.5 | 1.3 | 1.7 | 2x10^-7^ | 0.3819 |
| 9 | rs1701704 | 1.3 | 1.1 | 1.5 | 0.0002 |  | 1.4 | 1.2 | 1.7 | 2x10^-6^ | 0.3214 |
| 10 | rs2456973 | 1.3 | 1.1 | 1.5 | 0.0021 |  | 1.3 | 1.2 | 1.6 | 0.0001 | 0.5054 |
| 11 | rs1131017 | 1.2 | 1.1 | 1.4 | 0.0053 |  | 1.2 | 1.1 | 1.4 | 0.0058 | 0.9091 |
| 12 | rs12580100 | 1.0 | 0.8 | 1.3 | 0.7046 |  | 1.2 | 0.9 | 1.4 | 0.1836 | 0.1924 |
| 13 | rs11171739 | 1.2 | 1.0 | 1.3 | 0.0474 |  | 1.3 | 1.1 | 1.5 | 0.0012 | 0.6580 |
| 14 | rs2292239 | 1.2 | 1.1 | 1.4 | 0.0045 |  | 1.3 | 1.1 | 1.5 | 0.0003 | 0.8240 |
| 15 | rs2292238 | 1.3 | 1.1 | 1.5 | 0.0005 |  | 1.3 | 1.2 | 1.6 | 4x10^-5^ | 0.4086 |
| 16 | rs4759228 | 1.4 | 1.2 | 1.6 | 7x10^-6^ |  | 1.4 | 1.2 | 1.6 | 8x10^-5^ | 0.7796 |
| 17 | rs12810816 | 0.9 | 0.7 | 1.1 | 0.3473 |  | 0.8 | 0.6 | 1.0 | 0.0242 | 0.2438 |
| 18 | rs7311008 | 1.1 | 0.9 | 1.4 | 0.3423 |  | 1.4 | 1.1 | 1.7 | 0.0163 | 0.7434 |
| 19 | rs2291738 | 1.0 | 0.9 | 1.2 | 0.6031 |  | 1.1 | 0.9 | 1.2 | 0.4448 | 0.6384 |
