# Supplementary material for: Genetically Dependent ERBB3 Expression Modulates Antigen Presenting Cell Function and Type 1 Diabetes Risk
Source: PLoS One. 2010 Jul 26;5(7):e11789. doi: 10.1371/journal.pone.0011789 (PMC2909911; doi:10.1371/journal.pone.0011789)
Supplement: Table S4 — Results of multiple-SNP logistic regression analyses aimed at determining which SNPs had independent effects. (0.04 MB DOC) [file pone.0011789.s004.doc]

**Table S4**

| Method | Order Entered | SNP | Estimate | OR | LCL OR | UCL OR | p-value | BIC |
| --- | --- | --- | --- | --- | --- | --- | --- | --- |
| Chi-square Test | 1 | rs772921 | 0.4399 | 2.41 | 1.49 | 3.92 | 0.0004 | 3021.38 |
|  | 2 | rs1052165 | 0.2983 | 1.82 | 1.41 | 2.34 | 3.4x10^-6^ | 3012.10 |
|  | 3 | rs4759228 | 0.1963 | 1.48 | 1.03 | 2.13 | 0.0333 | 3018.02 |
|  | 4 | rs11171739 | -0.2202 | 0.64 | 0.43 | 0.97 | 0.0352 | 3023.45 |
| BIC | 1 | rs1701704 | 0.4050 | 2.25 | 1.74 | 2.91 | 6.2x10^-10^ | 3020.80 |
|  | 2 | rs1052165 | 0.2948 | 1.80 | 1.36 | 2.39 | 3.6x10^-5^ | 3011.28 |
| Lasso |  | rs1701704 | 0.1683 | 1.40 |  |  |  | 3020.80 |
|  |  | rs1052165 | 0.1051 | 1.23 |  |  |  | 3011.28 |
|  |  | rs773107 | 0.0351 | 1.07 |  |  |  | 3018.35 |
|  |  | rs4759228 | 0.0258 | 1.05 |  |  |  | 3024.60 |
|  |  | rs2292238 | 0.0166 | 1.03 |  |  |  | 3031.70 |
